# High-Quality Draft Genome Sequences of *Pantoea agglomerans* Isolates Exhibiting Antagonistic Interactions with Wheat Seed-Associated Fungi

**DOI:** 10.1128/genomeA.00511-16

**Published:** 2016-06-16

**Authors:** Jennifer Town, Tim J. Dumonceaux, Matt Links

**Affiliations:** a Agriculture and Agri-Food Canada, Saskatoon Research and Development Centre, Saskatoon, Saskatchewan, Canada; b Department of Veterinary Microbiology, University of Saskatchewan, Saskatoon, Saskatchewan, Canada; c Department of Computer Science, University of Saskatchewan, Saskatoon, Saskatchewan, Canada

## Abstract

*Pantoea agglomerans* isolates 3 and 4 were retrieved from the bacterial community associated with wheat seeds. These isolates differ in their pattern of growth antagonism toward *Alternaria* species. A comparison of the genome sequences of these two isolates revealed a high sequence identity with previously sequenced strains of *P. agglomerans*.

## GENOME ANNOUNCEMENT

Previous work examining the epiphytic microbiome of *Triticum* spp. and *Brassica* spp. identified strains of *Pantoea agglomerans* with differential growth antagonism phenotypes when cultured with fungi, such as *Alternaria* sp. and *Leptosphaeria maculans* (1). In particular, *P. agglomerans* isolate 4 inhibited the growth of these fungi, while isolate 3 did not exhibit this phenotype. To examine the mechanisms by which isolate 4 may inhibit the growth of fungal pathogens, we determined the genome sequences of these two isolates.

*P. agglomerans* isolates were grown at 28°C in a rotary shaker for 24 h in BBL Trypticase soy broth (Becton, Dickinson, Cockeysville, MD). Genomic DNA was purified from 1 ml of overnight culture using the Wizard genomic DNA (gDNA) extraction kit (Promega, Madison, WI). Sequencing was performed using Titanium Plus chemistry on a GS Junior platform (Roche Diagnostics, Laval, Quebec, Canada). Shotgun sequencing generated average read lengths of 458 bp (isolate 3) and 454 bp (isolate 4). In addition, an 8-kb insert paired-end sequencing run was performed on each isolate based on the paired-end rapid library preparation protocol for Titanium chemistry (Roche, March 2012), with modifications as described previously (2). The estimated pair distances were 6,111 ± 1,528 bp (isolate 3) and 6,346 ± 1,587 bp (isolate 4). Assembly of shotgun and paired-end sequencing runs for each genome using Newbler version 3.0 (454 Life Sciences) produced improved high-quality draft (3) sequences featuring 22× (isolate 3) and 26× (isolate 4) genome coverage. Each genome was assembled into 4 scaffolds and 6 scaffold contigs, with *N*_50_ scaffold sizes of 4,016,073 bp (isolate 3) and 3,947,245 bp (isolate 4). Sequence data were annotated using the Prokaryotic Genome Annotation Pipeline version 3.1 (NCBI) and using the Integrated Microbial Genomes portal (https://img.jgi.doe.gov/cgi-bin/mer/main.cgi).

The genomes of *P. agglomerans* isolates 3 and 4 contained 4,813,581 and 4,827,890 bp, respectively. Each genome contained 4,443 open reading frames (ORFs) but differed in the number of protein-coding genes (4,284 for isolate 3 and 4,305 for isolate 4). The genomes contained seven (isolate 3) or six (isolate 4) copies of the 16S rRNA-coding gene.

The sequences of the bacterial barcode *cpn60* (4) were identical between the two strains and 99 to 100% identical to those of *P. agglomerans*. While SpecI (5) could not assign either isolate to a species cluster, JSpecies (6) revealed that the two strains were very similar to one another and to *P. agglomerans* strain IG1 (7). Similarly, the strains shared very high (>98%) genomic average nucleotide identities (ANI) with many strains of *P. agglomerans*. Alignment of the scaffolds from the two isolates using NUCmer (8) indicated that the genomes were very similar to one another; however, a 109-kb scaffold was identified in isolate 4 that had no match in isolate 3. Annotation of this scaffold unique to isolate 4 identified several genes associated with conjugation, as well as genes encoding a CcdAB toxin-antitoxin system.

### Nucleotide sequence accession numbers.

These whole-genome shotgun projects have been deposited at DDBJ/ENA/GenBank under the accession numbers LVHW00000000 (isolate 3) and JPOT00000000 (isolate 4). The versions described in this paper are versions LVHW01000000 (isolate 3) and JPOT02000000 (isolate 4).
